# Identifying Acetylation Protein by Fusing Its PseAAC and Functional Domain Annotation

**DOI:** 10.3389/fbioe.2019.00311

**Published:** 2019-12-06

**Authors:** Wang-Ren Qiu, Ao Xu, Zhao-Chun Xu, Chun-Hua Zhang, Xuan Xiao

**Affiliations:** ^1^School of Information and Engineering, Jingdezhen Ceramic Institute, Jingdezhen, China; ^2^School of Life Science and Technology, University of Electronic Science and Technology of China, Chengdu, China

**Keywords:** acetylation, Random Forest, family and domain databases localization, post-translational modification, identification

## Abstract

Acetylation is one of post-translational modification (PTM), which often reacts with acetic acid and brings an acetyl radical to an organic compound. It is helpful to identify acetylation protein correctly for understanding the mechanism of acetylation in biological systems. Although many acetylation sites have been identified by high throughput experimental studies via mass spectrometry, there still are lots of acetylation sites need to be discovered. Computational methods have showed their power for identifying acetylation sites with informatics techniques which usually reduce experiment cost and improve the effectiveness and efficiency. In fact, if there is an approach can distinguish the acetylated proteins from the non-acetylated ones, it is no doubt a very meaningful and effective method for this issue. Here, we proposed a novel computational method for identifying acetylation proteins by extracting features from the conservation information of sequence via gray system model and KNN scores based on the information of functional domain annotation and subcellular localization. The authors have performed the 5-fold cross-validation on three datasets along with much analysis of features and the Relief feature selection algorithm. The obtained accuracies are all satisfactory, as the mean performance, the accuracy is 77.10%, the Matthew's correlation coefficient is 0.5457, and the AUC value is 0.8389. These works might provide useful insights for the related experimental validation, and further studies of other PTM process. For the convenience of related researchers, the web-server named “iACetyP” was established and is accessible at http://www.jci-bioinfo.cn/iAcetyP.

## Introduction

**Figure 1:**
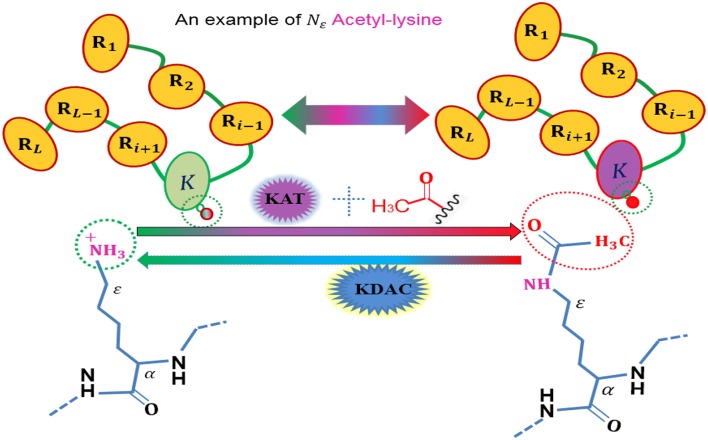
An illustration to show the acetylation protein.

To date, more than 450 unique protein modifications have been identified (Han et al., 2018), including phosphorylation, acetylation, ubiquitination, and sumoylation, which are regulatory mechanisms of cellular proteins with a number of biological functions, and also are very important for regulating the function of many prokaryotic and eukaryotic proteins (Yang et al., 2017). Among these post-translational modification (PTM), acetylation is a dynamic and highly conserved PTM (Figure 1) that plays a vital role in the regulating processes of diverse cellular. The role of acetylation in histones were first discovered in histones (Allfrey et al., 1964), and the first deacetylase activity was identified back in 1969 (Inoue and Fujimoto, 1969). Owing to its important involvement in some relevant biological processes, acetylation becomes one of the most important reversible protein posttranslational modifications, hence, more and more acetylated proteins are discovered with the help of high-throughput technologies. Thus, it is a piece of very interesting work to identify the potential acetylation sites for finding the underlying molecular mechanisms, and is helpful for basic bioresearch and drug development.

However, due to the importance and complexity of acetylation, identifying acetylation sites is a great challenge to fully understand the regulatory roles and the molecular mechanism of acetylation regulation. Actually, it is a time-consuming, expensive and labor-intensive process for purifying acetylation sites due to that the acetylation process is dynamic, rapid and reversible (Li et al., 2017; Yang et al., 2017). Fortunately, some studies had showed that experimental methods and computational models can be used to identify underlying PTMs sites (Hershko and Ciechanover, 1998; Haglund and Dikic, 2005; Tung and Ho, 2008; Radivojac et al., 2010), such as ubiquitination model of Radivojac et al. (2010); Zhao et al. (2011), and Cai et al. (2012), phosphorylation model of Ingrell et al. (2007); Yao et al. (2012, 2015); Chen et al. (2015); Li et al. (2015); Trost et al. (2015), and Xu et al. (2015), sumoylation model of Beauclair et al. (2015); Xu et al. (2016), and Han et al. (2018), acetylation model of Zhao et al. (2010); Wang et al. (2012); Hou et al. (2014), and Wuyun et al. (2016), and so on. Although these researchers did make much contribution to this issue, there is still a lot of room for improving the prediction quality. However, most of these efforts are on identifying some determinate PTMs sites for a given protein sequence, and few of computational method was proposed for distinguishing the acetylated proteins from the non-acetylated ones. This study was an attempt for the issue.

For a given protein represented with amino acid sequence, how to identify whether it may be one of some certain PTM proteins or may not? This may be the first step for identifying PTM sites and then is helpful and meaningful for basic research and drug development. In fact, we have made some preliminary exploration and attempt on identifying phosphorylated proteins. In Qiu et al. (2017a,b), we presented a method for identifying human phosphorylated proteins and a multi-label classifying model for different type of phosphorylated proteins with the help of the General PseAAC concept and gray system theory. Although the results are not so perfect, we still argue that the formulations and models can be applied to this issue, and it may be more powerful when some structure, function or localization information of proteins were added into the model. This site may be a fruitful opportunity for bioinformatics. For example, Gene Ontology (GO) (Ashburner et al., 2000) was proposed by Ashburner to reposit the concepts denoted as GO Terms that are associated to other gene products, and it has been widely used in describing the attributes for gene products (Agapito et al., 2016; Peng et al., 2016).

The dataset we used here was fully extracted from Uniprot (The UniProt, 2017). The present study tried to construct a classifying model for potential acetylation proteins by fusing the digital features which are come from its evolution information, Subcellular localization (noted as **SL**) (Nakai and Horton, 1999) information and functional domain annotation (noted as **FDA**) databases including GO (Harris et al., 2004), Pfam (Bateman et al., 1999), Smart (Letunic et al., 2004), InterPro (Hunter et al., 2009), PRINTS (Attwood et al., 2012), PROSITE (Sigrist et al., 2010), SUPFAM (Pandit et al., 2004). As for subcellular localization (Du et al., 2012), it was retrieved from the original UniProt database, which was reorganized by UniProt build-in hierarchical subcellular localization table. This paper proposed a new computational model for identifying potential acetylation proteins only on the basis of a query amino acid sequence by using its evolution information obtained with gray system model (Gray-PSSM) (Kaur and Raghava, 2004; Jones, 2007) and KNN scores calculated with the fuzzy distance by using its FDA and subcellular localization information. There are 80 amino acid sequence features extracted by incorporating the sequence evolution information were fused into PseAAC feature set and KNN scores, all of these features are combined according to different coefficients on the basis of its importance. To highlight the advantages of the proposed model, the model was trained and tested with three sub-datasets and cross-validations methods. In addition to some discussion of protein abovementioned features, some hypotheses for distinguishing acetylation proteins from non-acetylation ones were also depicted with the aid of training dataset.

## Materials and Methods

### Benchmark Dataset

It is fundamental and important that a stringent benchmark dataset be stablished for testing the proposed statistical predictor. Luckily, the UniProtKB/Swiss-Prot database is accepted by most of bioinformatics researchers, and has been using more and more widely. The data used in the current study to support this work are established on the basis of web http://www.uniprot.org.

In this study, we assume that our work is to identify whether an uncharacterized amino acid sequence is acetylation protein. As we known, the input sequence is comprised by amino acids and can be expressed as

(1) $$\begin{array}{r} \text{P}=P_{1}P_{2}P_{3}\cdots P_{i}\cdots P_{L} \end{array}$$

where *P*_*i*_ represents the *i*-th residue of amino acids sequence **P**, *L* is the length of **P**.

Here, we separate a benchmark dataset into a training dataset noted as *S*. Thus, the datasets can be formulated as:

(2) $$\begin{array}{r} {\text{S}}_{\text{all}}=S_{posi}\cup S_{nega}\;S_{nega}=S_{1}^{-}\cup S_{2}^{-}\cup S_{3}^{-} \end{array}$$

where *S*_*posi*_ is composed of the acetylation proteins, *S*_*nega*_ is composed of the non-acetylation proteins, $S_{i}^{-}\;\cap \;S_{j}^{-}\;=\;\emptyset \;(i\;\neq \;j;i,j\;=\;1,2,3)$. ∪ and ∩ represent the symbol for “set union” and “set intersection,” respectively.

The version of protein data used in the current study was released in May 2017. The positive dataset was generated according to the following criteria: (1) The potential acetylated proteins should be noted by anyone keyword of the set, i.e. {*N_acetylcysteine, N_acetylserine, N_acetylglutamate, N_acetylglycine, N_acetylproline, N_acetylthreonine, N_acetylvaline, N_acetylmethionine, N_acetyltyrosine, N2_acetylarginine, N6_acetyllysine, O_acetylserine, O_acetylthreonine*}. (2) The collected proteins are labeled by “Evidence” for the item of “Any assertion method.” (3) Only the proteins which consisting of 30 and more amino acid residues can be included, and the redundant proteins were removed with the threshold of 50% by using CD-HIT software.

The negative dataset was generated similar to the positive one except that those proteins should not be labeled none member of the mentioned above keyword-set. Since there are mass number of candidates here, we randomly collected negative datasets which have the balance samples size with positives.

Under the aforementioned standards, we obtained 2,925 protein samples, of which, the numbers of positive and negative samples are 725 and 2,175, respectively. In terms of Equation (2), we have 725 positive samples in *S*_*posi*_ and 2,175 negative samples in *S*_*nega*_. Here, we test the models with cross-validation on the three datasets with 1,450 samples, i.e., $S_{posi}\cup S_{1}^{-},S_{posi}\cup S_{2}^{-}\;$and $S_{posi}\cup S_{3}^{-}$, of which, the positive and negative ones are equal, i.e., 725 samples.

### Feature Construction

#### General Pseudo Amino Acid Composition (PseAAC)

Most of traditional machine-learning algorithms, such as Random Forest, SVM, and K nearest Neighbor, are not so powerful, the input should be vectors instead of sequence samples for biological issue. To overcome this problem, the researchers trying their best to improve the discrete or vector model by formulating the amino acids sequence into all kinds of pseudo amino acid composition (PseAAC), encoding method (Zhang et al., 2006; Chen et al., 2011; Shi et al., 2012; Jiao and Du, 2017) or other approaches.

Here, the proposed model followed the idea of PseAAC (Chou, 2011), and formulated an amino acids sequence **P** as:

(3) $$\begin{array}{r} P={\left[p_{1}\;p_{2}\;\cdots \;p_{u}\;\cdots \;p_{\text{N}}\right]}^{\text{T}} \end{array}$$

Here, the symbol **T** means the transpose operator for a matrix, *N* is an integer representing the number of features which depend on the method(s) used for extracting information from protein **P** (cf. Equation 1). *P* is a vector for representing amino acids sequence **P** and *p*_*i*_ (*i* = 1, 2, ⋯ , *N*) is the *i*th element of the vector. Below, we will describe how to extract functional domain annotation and subcellular localization information as well as pseudo amino acid composition, which are used in this study, from a query sequence to define the components for amino acids sequence **P**.

#### Protein Sample Formulation With KNN Score Based on FDA and Subcellular Localization (SL)

In addition to GO database, “Pfam,” “Smart,” “PROSITE,” “SUPFAM,” “InterPro,” and “PRINTS” are established according to cellular component, molecular function, biological process or some other characteristics. For example, the Pfam database is a large collected protein families generated by using hidden Markov models. SMART is abbreviation of Simple Modular Architecture Research Tool which can be used for research on the protein domains and architectures. PROSITE consists of entries describing the protein families, domains and functional sites as well as amino acid patterns and profiles. InterPro provides a functional analysis of protein sequences, and PRINTS also is a resource of detailed annotation for protein families in addition to a diagnostic tool for newly determined sequences. Subcellular localization feature is a key functional characteristic of potential gene products such as proteins, especially for plant.

Actually, in the GO database, proteins are clustered in a way in which their subcellular locations can be reflected fully. To incorporate more information, most of the approaches need to formulate a long list of the GO numbers, and a great part of the GO numbers make meaningless as a whole. In literatures (Gao et al., 2010; Yao et al., 2012), the authors show us that local sequence clusters often appear in the neighborhood of PTM sites for the reason that the same PTM family generally have some similarities in local sequences. As a better choice for depicting the character, K nearest neighbor score was proposed. To take advantage of such cluster information of GO and other FDA databases as well as subcellular localization for predicting acetylation proteins, for a given potential acetylation protein, we took the characteristics around the query neighborhood and extracted the KNN scores features from the training dataset containing both positive and negative samples. The algorithm is listed as follows.

Step 1. For a query protein sequence find its *k* nearest neighbors, which can be positive or negative samples, in the whole set according to local sequence similarity. For a given protein ***p***, $FDA_{j}(p)=\{N_{1}^{p,j},N_{2}^{p,j},\cdots ,N_{n_{p}}^{p,j}\}$ represents the keywords set of the *j*th FDA. The *j* (= 1, 2, …, 7, 8) represents “GO,” “Pfam,” “Smart,” “PROSITE,” “SUPFAM,” “InterPro,” “PRINTS,” or “subcellular localization,” respectively), ${\text{FDA}}_{j}(q)=\{N_{1}^{q,j},N_{2}^{q,j},\cdots ,N_{n_{q}}^{q,j}\}$ is the similar mean for protein ***q***. The similarity distance Dist_*j*_ (***p***, ***q***) between ***p*** and ***q*** can be defined as follows:

(4) $$\begin{array}{r} {\text{Dist}}_{j}\left(p,q\right)={\text{w}}_{1}\cdot \left(1-\frac{\left|{\text{FDA}}_{p}\left(j\right)\cap {\text{FDA}}_{q}\left(j\right)\right|}{\left|{\text{FDA}}_{p}\left(j\right)\cup {\text{FDA}}_{q}\left(j\right)\right|}\right)+{\text{w}}_{2}\cdot dist(p,q) \end{array}$$

Where ⋂, ⋃ and || are the operators “union,” “intersection,” and “norm” of the set theory, respectively. Here, || is defined as the number of its elements. *dist*(***p***, ***q*)** is the Euclidean distance on the basis of PseAAC. *w*_1_ and *w*_2_ are the weights of the two distances.

Step 2. A corresponding KNN feature is then extracted by calculating the KNN score, noted it as the percentage of acetylation proteins in its *k* nearest neighbors.

Step 3. To obtain diverse and enough properties of neighbors with KNN scores, the above two steps were repeated for different *k* values. For the *j*th member of FDA, the protein **P** can be formulated as:

(5) $$\begin{array}{r} P_{{\text{FDA}}_{j}}={\left[\varphi_{1}\left(j\right),\;\varphi_{2}\left(j\right),\cdots \;,\;\varphi_{K}\left(j\right)\;\right]}^{T} \end{array}$$

In this paper, the number of features is 50 and *k* was defined to be 0.1, 0.4, 0.7, …, 14.5 and 14.8 percent of the size of the involved dataset. In this way, 50 KNN scores were extracted as features for identifying acetylation proteins. To be more precisely, φ_1_(*j*) is the ratio of positive neighbors to whole concerned samples, i.e., 0.1 percent of the size of the training data set, φ_2_(*j*) is the ratio of positive neighbors to whole concerned samples whose value is the product of 0.004 and the size of the training data set, and so forth, when *K* = 50, φ_50_(*j*) is the ratio of positive neighbors to 14.8 percent of the size of the training data set.

In a word, a query protein sequence can be formulated with seven 50-Dimension vectors, i.e., ***P***_FDA_ = [***P***_FDA_**1**__, ***P***_FDA_**2**__, …, ***P***_FDA_**7**__], by using FDA database. Since Chou's pseudo amino acid composition (PseAAC) (Chou, 2001; Mondal and Pai, 2014) have showing so great powerful for identifying structure and function of protein, the proposed method took it into account according to the style of reference (Shen and Chou, 2008) (we select type 1 and let λ = 5). Thus, a given protein sequence can be expressed as 375-dimension vector, and these digital representations served as the input of the query protein for the prediction model.

### Operation Engine and Evaluation

#### Algorithms

**Figure 2:**
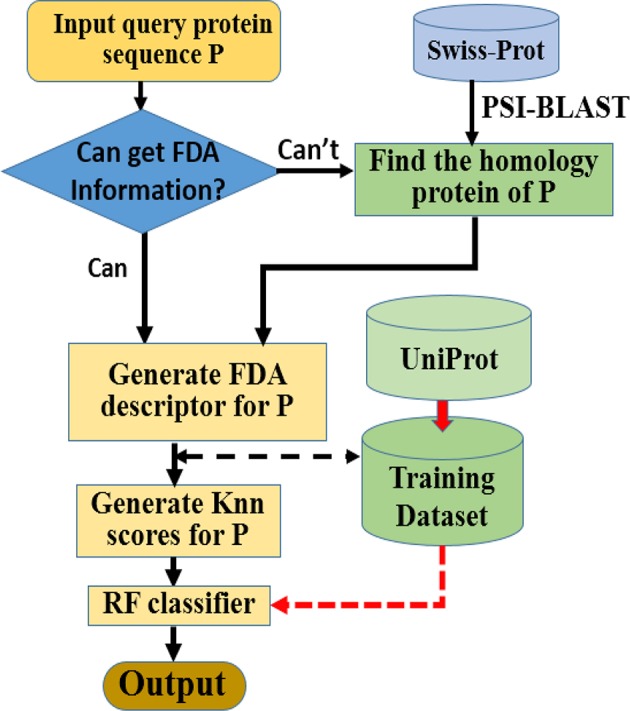
Flowchart of the proposed predictor.

Here we choose Random Forest as the operation engine as the predictor, and named the final predictor as “iAcet-PseFDA.” This name is an acronym created from its description, and Figure 2 would show how iAcet-PseFDA working.

As shown in Figure 2, the first step is to input the query amino acid sequence **P**. And then, the PSI-BLAST software was used to find the most similar protein to **P**, which is used to determine the most likely GO or other information of FDA set and generate the KNN scores with it. With the descriptor of **P**, the desired result can be obtained with the framework of Random Forest classifier trained on the benchmark.

#### Metrics and Test Method

The predictor iAcet-PseFDA was evaluated with cross-validation tests in the terms of following seven widely-accepted measurements: accuracy (or Acc, for short), Mathew's correlation coefficient abbreviated as Mcc, sensitivity (abbreviated as Sn, i.e., the fraction of the relevant documents that are successfully retrieved), specificity (i.e., Sep), Precision (i.e., Pre, a description of random errors), F-measure (or F-m, the harmonic mean of precision and recall), and G-mean. Since the area under the receiver operating characteristic curve (auROC, for short) is another important measurement of the performance of a given model, it was also calculated and plotted in this study. In view of the traits of validation method trait, cross-validation method was applied on three datasets for evaluating the proposed predictor.

## Results and Discussion

### Investigating the Performances of KNN Score of FDA Represent

**Figure 3:**
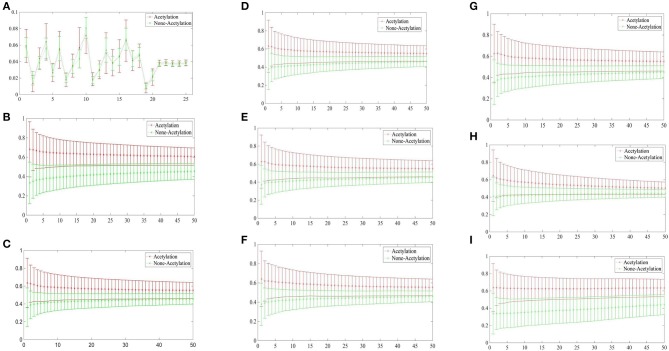
**(A)** Comparison of KNNScore-PAAC represents between acetylation proteins and non-acetylation proteins. **(B)** Comparison of KNNScore-GO represents between acetylation proteins and non-acetylation proteins. **(C)** Comparison of KNNScore-Pfam represents between acetylation proteins and non-acetylation proteins. **(D)** Comparison of KNNScore-Smart represents between acetylation proteins and non-acetylation proteins. **(E)** Comparison of KNNScore-Prosite represents between acetylation proteins and non-acetylation proteins. **(F)** Comparison of KNNScore-Supfam represents between acetylation proteins and non-acetylation proteins. **(G)** Comparison of KNNScore-InterPro represents between acetylation proteins and non-acetylation proteins. **(H)** Comparison of KNNScore-PRINTS represents between acetylation proteins and non-acetylation proteins. **(I)** Comparison of KNNScore-SL represents between acetylation proteins and non-acetylation proteins.

Figure 3 depicted the comparisons of the KNN scores of acetylation and non-acetylation proteins on all of the FDA features, and there really are some differences between the positive and negative samples. Figure 3A showed the comparison of PAAC represents between acetylation proteins and non-acetylation proteins, Figure 3B showed those of KNNScore-GO, and so forth, Figure 3I showed those of Subcellular localization. Overall, acetylation proteins gained obvious larger KNN scores than non-acetylation proteins on GO and Subcellular localization, and a little larger gap between the KNNScores of positive and negative datasets, all of the average KNN scores are nearly merged in 0.5 with the growth of features.

Specifically, for acetylation proteins with the view of GO evaluated on different sizes of nearest neighbors, the average values shown in Figure 3B are within 0.6–0.8, however, the average digits are within 0.2–0.4 for non-acetylation proteins. From the view of Subcellular localization as showed in Figure 3I, most of the average KNN scores of acetylation proteins are waved within 0.5–0.7 while those of non-acetylation proteins fluctuating around 0.4. From the view of Smart, Supfam, InterPro Pfam, Prosite and PRINTS as showed in Figures 3C–H, there are clearly gaps between the acetylation proteins and non-acetylation proteins, and the gaps are narrowing with the growth of KNNScores number.

We tested the eight kinds of features on the three datasets with RF, and the mean performances are depicted in the first 11th lines of Table 1, while the compared measurements obtained from the proposed model, in which the features were selected with Relief, are attached in the last line. As showed in the table, the features of Subcellular localization reached the best results with Acc is 73.95%, Mcc is 0.4843, Sn is 81.24%, Recall is 81.24%, F-measure is 75.72%, and G-mean is 73.59%. As regards for Sp and Precison, GO gained the best result which are 68.37 and 71.39%, respectively. Thus, the features of GO gained the second place. The other six performances are not satisfactory and worse than those of GO and Subcellular localization, all Accs of them are <0.7 except for GO and Subcellular localization. The results obtained with the enhanced model are discussed below.

**Table 1:** Mean performance comparison with different KNN score feature tested with RF.

	**Acc%**	**Mcc%**	**Sn%**	**Sp%**	**Pre%**	**F_m%**	**Gmean%**
PAAC	69.49	0.3909	73.01	65.98	68.22	70.53	69.40
GO	73.61	0.4754	78.85	68.37	71.39	74.90	73.39
Pfam	68.21	0.3647	70.99	65.43	67.27	69.07	68.14
Smart	67.01	0.3410	70.11	63.91	66.04	68.01	66.93
PROSITE	68.37	0.3679	70.85	65.89	67.52	69.14	68.31
SUPFAM	68.67	0.3738	71.17	66.16	67.79	69.44	68.62
InterPro	68.25	0.3658	71.40	65.10	67.17	69.22	68.18
PRINTS	66.11	0.3234	69.52	62.71	65.10	67.21	65.99
Subcellular localization	73.95	0.4843	81.24	66.67	70.91	75.72	73.59
All-MeanJK	74.64	0.4980	81.38	67.91	**71.78**	76.24	74.30
This paper	**77.55**	**0.5883**	**96.41**	**71.26**	52.79	**68.23**	**82.89**

^*^The bold value means the largest element of the column.

### Performance of Proposed Model

Based on the above discussions, we argue that the local amino acids surrounding acetylation sites, which have been verified, would share in similar pattern(s) with positive set on average as expected. These findings confirm that there are some acetylation-related clusters in acetylated proteins and hence may be used to distinguish them from the non-acetylation protein. Accordingly, the KNN scores were used to encode query sequence for predicting acetylation proteins in this study.

**Figure 4:**
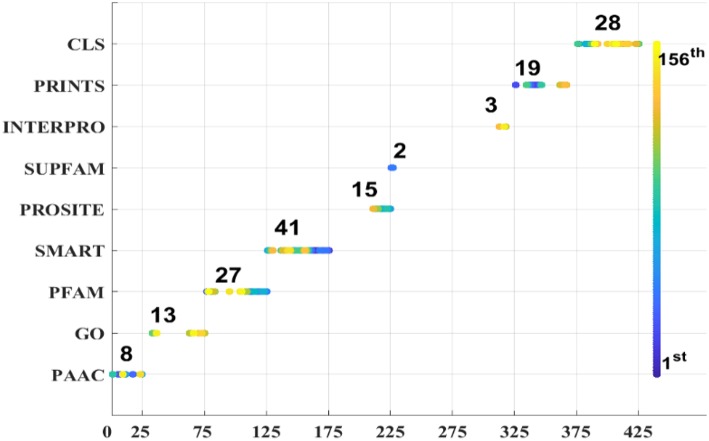
Features selected by Relief.

As we known, the Relief algorithm as a feature weighting algorithm was first proposed by Kira and Rendell (1992). In the algorithm, the features were allocated different weights in light of the relevance of characteristics and categories. The feature will be removed when its weight less than a threshold by this method. Since the combined features generated a high-dimensional vector, and the Relief method can rank the values of features, this work thus used Relief to reduce feature redundancy. With the help of Relief, we tested the predictor on different features sets and listed the mean performances in the last line of Table 1. The Acc is 77.55% which is better than 74.64%, the result obtained by using all of the eight features, and better than that of subcellular localization. The Relief model gained the better results according to the other seven measurements. Figure 4 depicted the selected features by Relief algorithm which containing 156 potential features (of which, there are 8 PSSM-gray features, 13 GO KNNScores, 27 for PFAM, 41 for SMART, 15 for PROSITE, 2 for SUPFAM, 3 for INTEPRO, 19 for PRINTS, and 28 for Subcellular localization KNNScores). From the figure, we can see that the importance of PAAC, SMART and PRINTS are obvious since a lot of features are noted as blue which means their rank in the selected feature set. The predictor obtains the best result at 156, which means there are 156 features were selected here, with Acc is 77.55%, Mcc is 0.5883, Sn is 96.41%, Sp is 71.26%, Precision is 52.79% which isn't the best performance unfortunately, Recall is 96.41%, F-measure is 68.23% and G-mean is 82.89%. These obtained results are better than anyone of Table 1.

**Figure 5:**
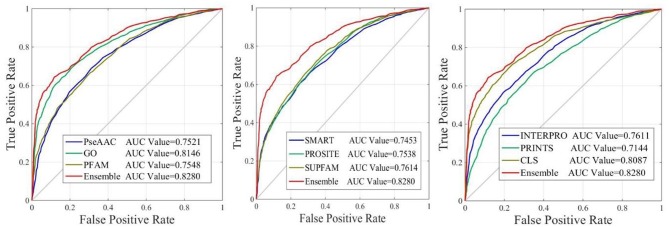
The ROC curves of predictor with different features.

The performance of iAcet-PseFDA was also depicted with ROC curves shown in Figure 5 in which the graphic lines are represent for GO, Subcellular localization and other Domain notations' KNNScores along with PseAAC's. As shown in first subfigure of Figure 5, the proposed model's AUC value is 0.8280 while those of PseAAC, GO, PFAM are 0.7521, 0.8146, 0.7548, respectively. Thus the proposed model obtained best result of the four methods. With similar analysis depicted in the last two subfigures of Figure 5, the AUC values of SMART, PROSITE, SUPFAM, INTERPRO, PRINTS, and Subcellular localization KNNScores are 0.7453, 0.7538, 0.7614, 0.7611, 0.7144, and 0.8087, respectively. In conclusion, all of the values are <0.8280, and there still are gaps between them and that of the proposed model. It shows that the feature set enhanced with Relief would obtain more satisfactory results than those of the independent FDA features.

## Conclusion

In order to detect acetylation proteins, this study developed a method on the basis of Random Forest algorithm and Relief. Our approach considered information of sequence conservation extracted by PSI-BLAST besides with PseACC. The involved features are extracted from the sequence conservation information and “GO,” “Pfam,” “Smart,” “PROSITE,” “SUPFAM,” “InterPro,” “PRINTS” and Subcellular localization information of the given query amino acid sequence. This work may cope with the expensive and time-consuming process of identifying acetylation proteins because that the features only incorporated the sequence conservation via gray system model and Knn scores based on FDA databases. All of these processes only need computational model instead of any physical chemistry experiment.

Also, our result manifested that it appears that using FDAs is essential for the prediction of acetylation functional class, which had been reported in previous research (Qiu et al., 2016a,b, 2017b), and the information related to subcellular is also important for identifying the PTM proteins. As the growing demand of verification of acetylation sites, we argue that more effort should be input in developing organism-specific predictors for this issue. The reason for presenting the model here then is for the improving the predictor used in similar research, and it may be helpful for those researchers who would like to deal with bioinformatics problems with computational models. In addition, the involved features may provide important clues of the acetylation mechanism and guide the related experimental validations.

Additionally, a web-server has been established at http://www.jci-bioinfo.cn/iAcetyP which is user-friendly and convenient for the researchers who are working in distinguishing acetylated proteins from non-acetylated proteins.

## Data Availability Statement

Publicly available datasets were analyzed in this study. This data can be found here: http://www.uniprot.org/.

## Author Contributions

AX and Z-CX carried out the extraction of annotation features, model construction, model training, and evaluation, also drafted the related initial manuscript version. C-HZ carried out the preparation of the data, GO enrichment, subcellular localization analysis, comparison with deep learning tool, and drafted the subsequent manuscript. XX led this project and guided this work and shared his idea to implement and discussion. All authors involved in discussion and conclusion section.

### Conflict of Interest

The authors declare that the research was conducted in the absence of any commercial or financial relationships that could be construed as a potential conflict of interest. The handling editor declared a shared affiliation, though no other collaboration, with one of the authors W-RQ.

## References

[B1] Agapito G. Milano M. Guzzi P. H. Cannataro M. (2016). Extracting cross-ontology weighted association rules from gene ontology annotations. IEEE-ACM Trans. Comput. Biol. Bioinform. 13, 197–208. 10.1109/TCBB.2015.2462348 27045823

[B2] Allfrey V. G. Faulkner R. Mirsky A. E. (1964). Acetylation and methylation of histones and their possible role in the regulation of rna synthesis. Proc. Natl. Acad. Sci. U.S.A. 51, 786–794. 10.1073/pnas.51.5.786 14172992 PMC300163

[B3] Ashburner M. Ball C. A. Blake J. A. Botstein D. Butler H. Cherry J. M.. (2000). Gene ontology: tool for the unification of biology. Nat. Genet. 25, 25–29. 10.1038/75556 10802651 PMC3037419

[B4] Attwood T. K. Coletta A. Muirhead G. Pavlopoulou A. Philippou P. B. Popov I.. (2012). The PRINTS database: a fine-grained protein sequence annotation and analysis resource–its status in 2012. Database (Oxford). 2012:bas019. 10.1093/database/bas019 22508994 PMC3326521

[B5] Bateman A. Birney E. Durbin R. Eddy S. R. Finn R. D. Sonnhammer E. L. (1999). Pfam 3.1: 1313 multiple alignments and profile HMMs match the majority of proteins. Nucleic Acids Res. 27, 260–262. 10.1093/nar/27.1.260 9847196 PMC148151

[B6] Beauclair G. Bridier-Nahmias A. Zagury J. F. Saïb A. Zamborlini A. (2015). JASSA: a comprehensive tool for prediction of SUMOylation sites and SIMs. Bioinformatics 31, 3483–3491. 10.1093/bioinformatics/btv403 26142185

[B7] Cai Y. Huang T. Hu L. Shi X. Xie L. Li Y. (2012). Prediction of lysine ubiquitination with mRMR feature selection and analysis. Amino Acids 42, 1387–1395. 10.1007/s00726-011-0835-0 21267749

[B8] Chen X. Shi S. P. Suo S. B. Xu H. D. Qiu J. D. (2015). Proteomic analysis and prediction of human phosphorylation sites in subcellular level reveal subcellular specificity. Bioinformatics 31, 194–200. 10.1093/bioinformatics/btu598 25236462

[B9] Chen Z. Chen Y. Z. Wang X. F. Wang C. Yan R. X. Zhang Z. (2011). Prediction of ubiquitination sites by using the composition of k-spaced amino acid Pairs. PLoS ONE 6:e22930. 10.1371/journal.pone.0022930 21829559 PMC3146527

[B10] Chou K. C. (2001). Prediction of protein cellular attributes using pseudo-amino acid composition. Proteins 43, 246–255. 10.1002/prot.1035 11288174

[B11] Chou K. C. (2011). Some remarks on protein attribute prediction and pseudo amino acid composition. J. Theor. Biol. 273, 236–247. 10.1016/j.jtbi.2010.12.024 21168420 PMC7125570

[B12] Du P. Tian Y. Yan Y. (2012). Subcellular localization prediction for human internal and organelle membrane proteins with projected gene ontology scores. J. Theor. Biol. 313, 61–67. 10.1016/j.jtbi.2012.08.016 22960368

[B13] Gao J. Thelen J. J. Dunker A. K. Xu D. (2010). Musite, a tool for global prediction of general and kinase-specific phosphorylation sites. Mol. Cell. Proteomics 9, 2586–2600. 10.1074/mcp.M110.001388 20702892 PMC3101956

[B14] Haglund K. Dikic I. (2005). Ubiquitylation and cell signaling. EMBO J. 24, 3353–3359. 10.1038/sj.emboj.7600808 16148945 PMC1276169

[B15] Han Z. J. Feng Y. H. Gu B. H. Li Y. M. Chen H. (2018). The post-translational modification, SUMOylation, and cancer (Review). Int. J. Oncol. 52, 1081–1094. 10.3892/ijo.2018.4280 29484374 PMC5843405

[B16] Harris M. A. Clark J. Ireland A. Lomax J. Ashburner M. Foulger R.. (2004). The Gene Ontology (GO) database and informatics resource. Nucleic Acids Res. 32, D258–D261. 10.1093/nar/gkh036 14681407 PMC308770

[B17] Hershko A. Ciechanover A. (1998). The ubiquitin system. Annu. Rev. Biochem. 67, 425–479. 10.1146/annurev.biochem.67.1.425 9759494

[B18] Hou T. Zheng G. Zhang P. Jia J. Li J. Xie L.. (2014). LAceP: lysine acetylation site prediction using logistic regression classifiers. PLoS ONE 9:e89575. 10.1371/journal.pone.0089575 24586884 PMC3930742

[B19] Hunter S. Apweiler R. Attwood T. K. Bairoch A. Bateman A. Binns D.. (2009). InterPro: the integrative protein signature database. Nucleic Acids Res. 37, D211–D215. 10.1093/nar/gkn785 18940856 PMC2686546

[B20] Ingrell C. R. Miller M. L. Jensen O. N. Blom N. (2007). NetPhosYeast: prediction of protein phosphorylation sites in yeast. Bioinformatics. 23, 895–897. 10.1093/bioinformatics/btm020 17282998

[B21] Inoue A. Fujimoto D. (1969). Enzymatic deacetylation of histone. Biochem. Biophys. Res. Commun. 36, 146–150. 10.1016/0006-291X(69)90661-5 5796748

[B22] Jiao Y. S. Du P. F. (2017). Predicting protein submitochondrial locations by incorporating the positional-specific physicochemical properties into Chou's general pseudo-amino acid compositions. J. Theor. Biol. 416, 81–87. 10.1016/j.jtbi.2016.12.026 28077336

[B23] Jones D. T. (2007). Improving the accuracy of transmembrane protein topology prediction using evolutionary information. Bioinformatics 23, 538–544. 10.1093/bioinformatics/btl677 17237066

[B24] Kaur H. Raghava G. P. (2004). A neural network method for prediction of beta-turn types in proteins using evolutionary information. Bioinformatics 20, 2751–2758. 10.1093/bioinformatics/bth322 15145798

[B25] Kira K. Rendell L. A. (1992). The feature selection problem: traditional methods and a new algorithm, in Tenth National Conference on Artificial Intelligence (San Jose, CA: San Jose Convention Center).

[B26] Letunic I. Copley R. R. Schmidt S. Ciccarelli F. D. Doerks T. Schultz J.. (2004). SMART 4.0: towards genomic data integration. Nucleic Acids Res. 32, D142–D144. 10.1093/nar/gkh088 14681379 PMC308822

[B27] Li C. Choi H. P. Wang X. Wu F. Chen X. Lü X.. (2017). Post-translational modification of human histone by wide tolerance of acetylation. Cells 6:34. 10.3390/cells6040034 29023412 PMC5753069

[B28] Li Z. Wu P. Zhao Y. Liu Z. Zhao W. (2015). Prediction of serine/threonine phosphorylation sites in bacteria proteins. Adv. Exp. Med. Biol. 827, 275–285. 10.1007/978-94-017-9245-5_16 25387970

[B29] Mondal S. Pai P. P. (2014). Chou's pseudo amino acid composition improves sequence-based antifreeze protein prediction. J. Theor. Biol. 356, 30–35. 10.1016/j.jtbi.2014.04.006 24732262

[B30] Nakai K. Horton P. (1999). PSORT: a program for detecting sorting signals in proteins and predicting their subcellular localization. Trends Biochem. Sci. 24, 34–36. 10.1016/S0968-0004(98)01336-X 10087920

[B31] Pandit S. B. Bhadra R. Gowri V. S. Balaji S. Anand B. Srinivasan N. (2004). SUPFAM: a database of sequence superfamilies of protein domains. BMC Bioinformatics 5:28. 10.1186/1471-2105-5-28 15113407 PMC394316

[B32] Peng J. Wang T. Wang J. Wang Y. Chen J. (2016). Extending gene ontology with gene association networks. Bioinformatics 32, 1185–1194. 10.1093/bioinformatics/btv712 26644414

[B33] Qiu W. R. Sun B. Q. Xiao X. Xu D. Chou K. C. (2017a). iPhos-PseEvo: identifying human phosphorylated proteins by incorporating evolutionary information into general PseAAC via grey system theory. Mol. Inform. 36:1600010. 10.1002/minf.201600010 28488814

[B34] Qiu W. R. Sun B. Q. Xiao X. Xu Z. C. Chou K. C. (2016b). iPTM-mLys: identifying multiple lysine PTM sites and their different types. Bioinformatics 32, 3116–3123. 10.1093/bioinformatics/btw380 27334473

[B35] Qiu W. R. Xiao X. Xu Z. C. Chou K. C. (2016a). iPhos-PseEn: identifying phosphorylation sites in proteins by fusing different pseudo components into an ensemble classifier. Oncotarget 7, 51270–51283. 10.18632/oncotarget.9987 27323404 PMC5239474

[B36] Qiu W. R. Zheng Q. S. Sun B. Q. Xiao X. (2017b). Multi-iPPseEvo: a multi-label classifier for identifying human phosphorylated proteins by incorporating evolutionary information into Chou's general PseAAC via grey system theory. Mol. Inform. 36:1600085. 10.1002/minf.201600085 27681207

[B37] Radivojac P. Vacic V. Haynes C. Cocklin R. R. Mohan A. Heyen J. W.. (2010). Identification, analysis, and prediction of protein ubiquitination sites. Proteins 78, 365–380. 10.1002/prot.22555 19722269 PMC3006176

[B38] Shen H. B. Chou K. C. (2008). PseAAC: a flexible web server for generating various kinds of protein pseudo amino acid composition. Anal. Biochem. 373, 386–388. 10.1016/j.ab.2007.10.012 17976365

[B39] Shi S. P. Qiu J. D. Sun X. Y. Suo S. B. Huang S. Y. Liang R. P. (2012). A method to distinguish between lysine acetylation and lysine methylation from protein sequences. J. Theor. Biol. 310, 223–230. 10.1016/j.jtbi.2012.06.030 22796329

[B40] Sigrist C. J. Cerutti L. de Castro E. Langendijk-Genevaux P. S. Bulliard V. Bairoch A.. (2010). PROSITE, a protein domain database for functional characterization and annotation. Nucleic Acids Res. 38, D161–D166. 10.1093/nar/gkp885 19858104 PMC2808866

[B41] The UniProt C. (2017). UniProt: the universal protein knowledgebase. Nucleic Acids Res. 45, D158–D169. 10.1093/nar/gkw1099 27899622 PMC5210571

[B42] Trost B. Napper S. Kusalik A. (2015). Case study: using sequence homology to identify putative phosphorylation sites in an evolutionarily distant species (honeybee). Brief. Bioinform. 16, 820–829. 10.1093/bib/bbu040 25380664

[B43] Tung C. W. Ho S. Y. (2008). Computational identification of ubiquitylation sites from protein sequences. BMC Bioinform. 9:310. 10.1186/1471-2105-9-310 18625080 PMC2488362

[B44] Wang L. Du Y. Lu M. Li T. (2012). ASEB: a web server for KAT-specific acetylation site prediction. Nucleic Acids Res. 40, W376–W379. 10.1093/nar/gks437 22600735 PMC3394258

[B45] Wuyun Q. Zheng W. Zhang Y. Ruan J. Hu G. (2016). Improved species-specific lysine acetylation site prediction based on a large variety of features set. PLoS ONE 11:e0155370. 10.1371/journal.pone.0155370 27183223 PMC4868276

[B46] Xu X. Li A. Wang M. (2015). Prediction of human disease-associated phosphorylation sites with combined feature selection approach and support vector machine. IET Syst. Biol. 9, 155–163. 10.1049/iet-syb.2014.0051 26243832 PMC8687269

[B47] Xu Y. Ding Y. X. Deng N. Y. Liu L. M. (2016). Prediction of sumoylation sites in proteins using linear discriminant analysis. Gene 576(1 Pt 1), 99–104. 10.1016/j.gene.2015.09.072 26432000

[B48] Yang Y. Tong M. Bai X. Liu X. Cai X. Luo X.. (2017). Comprehensive proteomic analysis of lysine acetylation in the foodborne pathogen *Trichinella spiralis*. Front. Microbiol. 8:2674. 10.3389/fmicb.2017.02674 29375535 PMC5768625

[B49] Yao Q. Gao J. Bollinger C. Thelen J. J. Xu D. (2012). Predicting and analyzing protein phosphorylation sites in plants using musite. Front. Plant Sci. 3:186. 10.3389/fpls.2012.00186 22934099 PMC3423629

[B50] Yao Q. Schulze W. X. Xu D. (2015). Phosphorylation site prediction in plants. Methods Mol. Biol. 1306, 217–228. 10.1007/978-1-4939-2648-0_17 25930706

[B51] Zhang Z. H. Wang Z. H. Zhang Z. R. Wang Y. X. (2006). A novel method for apoptosis protein subcellular localization prediction combining encoding based on grouped weight and support vector machine. FEBS Lett. 580, 6169–6174. 10.1016/j.febslet.2006.10.017 17069811

[B52] Zhao C. Liu H. Li J. Deng Y. Shi T. (2010). Nucleosome structure incorporated histone acetylation site prediction in Arabidopsis thaliana. BMC Genom. 11(Suppl 2):S7. 10.1186/1471-2164-11-S2-S7 21047388 PMC2975415

[B53] Zhao X. Li X. Ma Z. Yin M. (2011). Prediction of lysine ubiquitylation with ensemble classifier and feature selection. Int. J. Mol. Sci. 12, 8347–8361. 10.3390/ijms12128347 22272076 PMC3257073

